# Elevated Nitrogen Priming Induced Oxinitro-Responses and Water Deficit Tolerance in Rice

**DOI:** 10.3390/plants10020381

**Published:** 2021-02-17

**Authors:** Kamolchanok Umnajkitikorn, Mitsutaka Fukudome, Toshiki Uchiumi, Neung Teaumroong

**Affiliations:** 1 School of Crop Production Technology, Institute of Agricultural Technology, Suranaree University of Technology, Nakhon Ratchasima 30000, Thailand; 2 Graduate School of Science and Engineering, Kagoshima University, 1-21-35 Korimoto, Kagoshima 890-0065, Japan; fukudome@nibb.ac.jp (M.F.); uttan@sci.kagoshima-u.ac.jp (T.U.); 3 Division of Symbiotic Systems, National Institute for Basic Biology, Myodaiji, Okazaki, Aichi 444-8585, Japan; 4 School of Biotechnology, Institute of Agricultural Technology, Suranaree University of Technology, Nakhon Ratchasima 30000, Thailand; neung@sut.ac.th

**Keywords:** rice, nitrogen, water stress, drought, antioxidant, reactive oxygen species, reactive nitrogen species

## Abstract

Under water deficit conditions, the essential macronutrient nitrogen becomes limited as a result of reduced dissolved nitrogen and root nitrogen uptake. An elevated nitrogen level might be able to mitigate these effects, integrated with the idea of using nitric oxide as abiotic stress tolerant inducers. In this study, we evaluated the potential of using elevated nitrogen priming prior to water shortage to mitigate plant stress through nitric oxide accumulation. We grew rice plants in 300 mg L^−1^ nitrogen for 10 weeks, then we primed plants with four different nitrogen concentrations: 100, 300 (control), 500 and 1000 mg L^−1^ nitrogen prior to inducing water deficit conditions. Plants primed with 500 mg L^−1^ nitrogen possessed a higher photosynthetic rate, relative water content, electrolyte leakage and lipid peroxidation under water deficit conditions, compared to control plants. The induction of water deficit tolerance was supported with the activation of antioxidant defense system, induced by the accumulation of nitric oxide in leaves and roots of rice plants. We originally demonstrated the accumulation of nitric oxide in leaves of rice plants. The elevated nitrogen priming can be used to enhance water deficit tolerance in irrigated paddy fields, instead of nitric oxide donors.

## 1. Introduction

Water deficit conditions cause widespread crop loss globally due to abiotic stress. By the end of the 21st century, drought and water deficit stress may account for more than 70 percent of crop loss below optimal productivity [1]. There are many methods to cope with stresses from water deficit conditions: breeding strategies, cultural practices, and irrigation approaches [2,3]. Drought stress induces mature and young leaf senescence. The primary site of damage during stress is the chloroplasts, where more than 70% of the leaf nitrogen (N) is sequestrated. In many annual crop species, chloroplast degradation requires a main strategy to cope with drought stress—known as an escape strategy [4]. This strategy aims to utilize the accumulated nutrients, particularly nitrogen and energy for acclimation and survival during stress episodes [5].

Under water deficit conditions, the loss of transpiration and cell turgor pressure lead to a reduction in uptake and transport of nitrogen, silicon, magnesium and calcium and other important nutrients [6]. Water deficit does not only limit nitrogen uptake but also restricts nitrogen assimilation through the inhibition of enzymes involved in nitrogen metabolism, such as nitrate reductase and glutamine synthetase [7]. Leaf yellowing commonly appears as the consequence of water deficit due to low chlorophyll biosynthesis and high chloroplast and chlorophyll degradation [5].

Nitric oxide (NO) has been proposed as a regulator of the antioxidant defense system during stress conditions [8,9,10,11]. Cai et al. 2015 found that the activity of nitric oxide synthase (NOS) and the accumulation of NO in rice plants were activated under salinity and drought stress [9]. Transgenic rice plants with overexpression of rat neuronal NO synthase (nNOS) showed an improvement in drought and salt tolerance [9]. External application of nitric oxide (NO) donor S-nitroso-N-acetylpenicillamine (SNAP) also alleviated the damage from salinity stress on chickpea (Cicer arietinum L.) plants [11]. The application of sodium nitroprussiate (SNP) (NO donor) also improved the chilling tolerance of winter wheat via the accumulation of fructan [12]. Spraying rice seedlings with SNP also enhanced drought tolerance, maintained leaf water potential, enhanced the antioxidant defense system and improved the stability of membranes [13]. However, NO donors or the transgenic approach are costly and inapplicable in many parts of the world. We were eager to search for a simplified method to utilize NO-triggered tolerant mechanisms. NO can be generated as a byproduct from a reaction catalyzed with nitrate reductase (NR) [14,15]. NR-generated NO had a pivotal role in lateral root formation and nitrogen uptake under partial nitrate nutrition in rice [16]. A high level of nitrogen application therefore becomes an interesting option due to the fact that farmers can access and afford nitrogen fertilizer.

Long-term application of high amounts of chemical N fertilizer lessened the water and nutrient use efficiency as well as inducing environmental problems and sensitivity to insects and diseases [17,18]. However, with moderate drought stress an appropriate level of N application can benefit the growth and yield of rice plants. Applying nitrogen can induce leaf production and thus accelerate transpiration and drought stress severity, and elevated nitrogen application levels may further stimulate rice shoot/root growth, promote photosynthesis, transpiration and yield [19,20]. High nitrogen levels also enhanced plasticity of roots, allowing plants to extract more of the water available from the soil [21]. Moreover, high N application induced a reduction in mesophyll conductance (g_m_), which limits photosynthetic capacity and N-use efficiency [17]. Some recent studies have also shown that a high level of N in growing media enhanced the antioxidant defense system and some nitrogen assimilation enzymes, such as glutamine synthetase (GS) and glutamate synthase (GOGAT) but decreased N-use efficiency [22]. Although the effect of a large amount of N in growing media or in soil has long been seen to promote photosynthesis and N assimilation in various crop plants, regulation of high N levels triggering drought tolerance through NO signaling has never been investigated.

We therefore hypothesized that if we increased the nitrogen concentration in the fertilizer for only a short-period of time (priming), NO accumulation would be activated. Thus, the antioxidant defense system would also be activated. An addition of nitrogen could delay protein and chlorophyll degradation during a short period of water scarcity. In this study we aim to identify the appropriate level of N for priming treatment prior to stress episodes and to assess the mechanisms of nitrogen induced water deficit tolerance.

## 2. Results

### 2.1. Elevated Nitrogen Priming Enhanced Photosynthesis and Leaf Growth under Water Deficit Conditions

**Figure 1:**
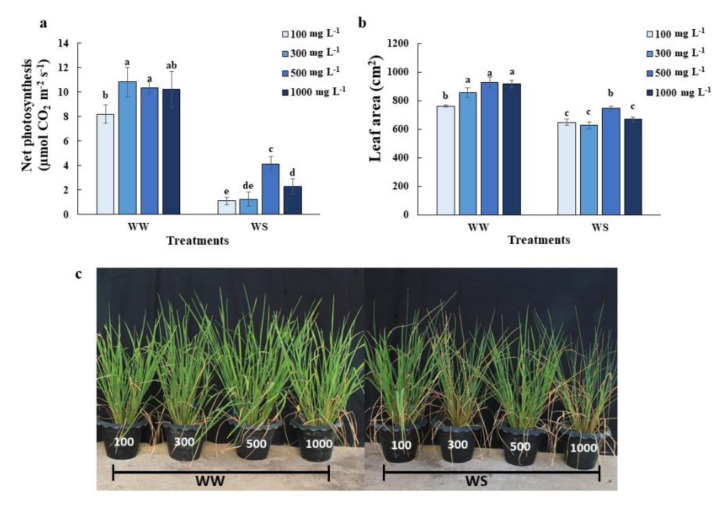
Growth and photosynthesis responses of rice plants to different nitrogen concentration priming. (**a**) Net photosynthesis of the youngest fully expanded leaves of the greenhouse-grown 11-week old rice plants after maintaining relative soil moisture content at 10–15% for 7 days (WS), compared with those under well-watered conditions (WW). (**b**) Leaf area of plants subjected to different level of nitrogen priming. (**c**) Plant growth under different level of nitrogen priming. The values shown are the Mean ± SE (n = 6 and 10, respectively). The different letters above the bars indicate significant differences by one-way ANOVA and Duncan’s test (*p* ≤ 0.05).

An elevated nitrogen level is well-known for promoting plant growth. However, the effects of an elevated nitrogen level for short term application (priming) on photosynthesis and growth have not been investigated. Rice plants primed with an elevated N level (500 mg L^−1^ and 1000 mg L^−1^ N) maintained higher photosynthetic activity (Figure 1a), but only the 500 mg L^−1^ N promoted rice plant growth as indicated by increasing leaf area under water deficit conditions (Figure 1b,c) when compared to the control plants (at 300 mg L^−1^ N). Elevated nitrogen priming failed to alter the net photosynthetic rate or leaf area of rice plants under well-watered conditions. Low N level priming had lower photosynthetic activity and growth under well-watered conditions (Figure 1).

### 2.2. Elevated Nitrogen Priming Promoted Relatively Higher Leaf Relative Water Content (RWC) under Water Deficit Conditions

**Figure 2:**
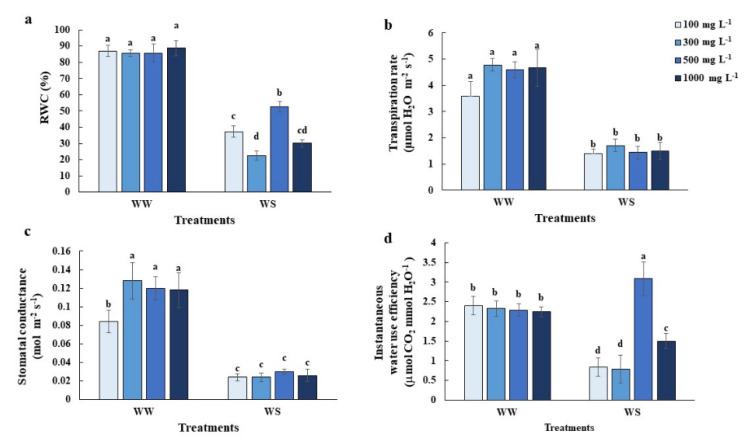
Physiological responses associated with plant water status. (**a**) relative leaf water content (RWC), (**b**) stomatal conductance, (**c**) transpiration rate and (**d**) instantaneous water use efficiency of the youngest fully expanded leaves of the greenhouse-grown 11-week old rice plants after maintaining relative soil moisture content at 10–15% for 7 days, compared with those under well-watered conditions (WW). Values are the Mean ± SE (n = 6). The different letters above the bars indicate significant differences by one-way ANOVA and Duncan’s test (*p* ≤ 0.05).

Elevated N-primed rice had higher RWC under water deficit conditions than normal N and lower N-primed rice (Figure 2a). Stomata conductance and transpiration rate from different priming nitrogen concentrations under water deficit conditions showed no significant differences. However, under well-watered conditions N-primed plants showed significantly lower stomata conductance (Figure 2b,c). A dramatically increased instantaneous water use efficiency occurred only in rice plants subjected to 500 mg L^−1^ N-priming treatment (Figure 2d).

### 2.3. Elevated Nitrogen Priming Alleviated Chlorophyll and Protein Degradation as Well as Cell Damage under Water Deficit Conditions

**Figure 3:**
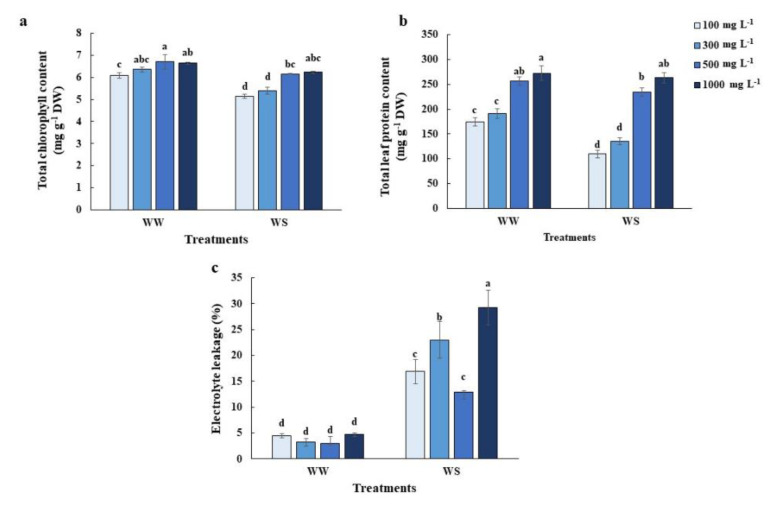
Biochemical responses associated with leaf senescence: (**a**) Total chlorophyll content, (**b**) total leaf protein content and (**c**) electrolyte leakage of the youngest fully expanded leaves of the greenhouse-grown 11-week old rice plants after maintaining relative soil moisture content at 10–15% for 7 days (WS), compared with those under well-watered conditions (WW). Values shown are the Mean ± SE (n = 4, 6 and 4, respectively). The different letters above the bars indicate significant differences by one-way ANOVA and Duncan’s test (*p* ≤ 0.05).

The elevated N-primed leaf maintained significantly higher chlorophyll and protein content under water deficit conditions (Figure 3a,b) but had only significantly higher protein content under well-watered conditions (Figure 3b). Electrolyte leakage (EL) was lower in the 500 mg L^−1^ N-primed plants and low N-primed plants (Figure 3c). However, excessive N (1000 mg L^−1^ N) had significantly higher electrolyte leakage (Figure 3c).

### 2.4. Elevated Nitrogen Priming Enhanced Antioxidant Defense Mechanisms under Water Deficit Conditions

**Figure 4:**
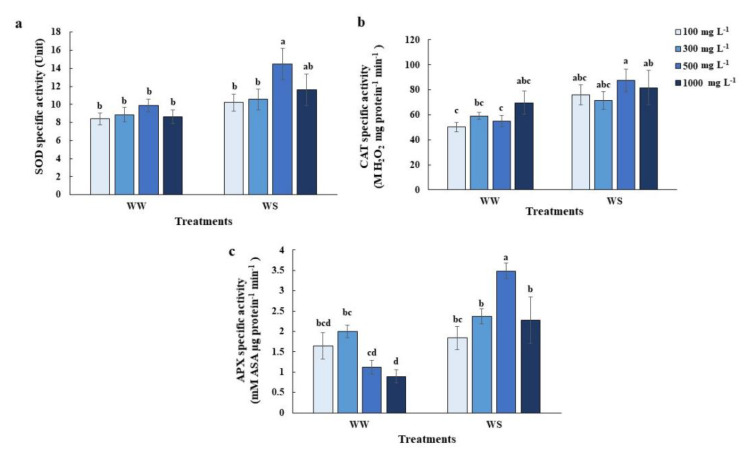
Enzymatic reactive oxygen species (ROS)-scavenging antioxidant responses: (**a**) Superoxide dismutase (SOD), (**b**) catalase (CAT) and (**c**) ascorbate peroxidase (APX) specific activities of the youngest fully expanded leaves of the 5 greenhouse-grown 11-week old rice plants after maintaining relative soil moisture content at 10–15% for 7 days (WS), compared with those under well-watered conditions (WW). Values are the Mean ± SE (n = 4). The different letters above the bars indicate significant differences by one-way ANOVA and Duncan’s test (*p* ≤ 0.05).

To elucidate the mechanisms of elevated-N priming in delaying leaf senescence, we investigated the antioxidant defense system. Under well-watered conditions, SOD and CAT activity were the same between different N priming treatments (Figure 4a,b), while APX activity was slightly lower 100 mg L^−1^ and 500 mg L^−1^ N-primed plants and significantly lower in 1000 mg L^−1^ N-primed plants, compared to nonprimed (300 mg L^−1^ N) plants (Figure 4c). In contrast, under water deficit conditions, SOD and APX activities significantly increased in plants primed with 500 mg L^−1^ N, compared to control (Figure 4a,b). However, CAT activity in elevated N-primed plants significantly increased under water deficit conditions, compared to control (300 mg L^−1^ N) under well-watered conditions (Figure 4b).

### 2.5. Nitric Oxide Accumulation in Elevated Nitrogen Primed-Plants Alleviated Reactive Oxygen Species (ROS) Accumulation under Water Deficit Conditions

**Figure 5:**
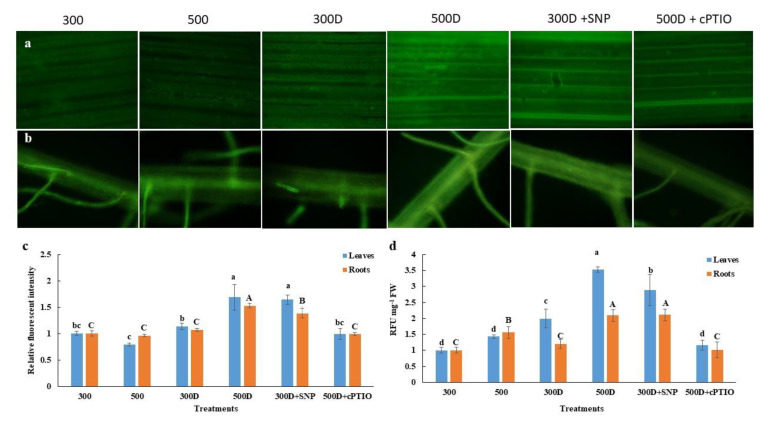
Reactive nitrogen species accumulation responses. The epifluorescent images of rice (**a**) leaves and (**b**) roots after 1-h incubation in 20 μM DAF-FM DA. The plants were subjected to the different NO-associated treatments for 3. days: 300 mg L^−1^ N-primed (300C); 500 mg L^−1^ N-primed (500C); 300 mg L^−1^ N-primed with PEG water deficit induction (300D); 500 mg L^−1^ N-primed with PEG water deficit induction (500D); 300 mg L^−1^ N-primed with PEG water deficit induction + Nitric oxide donor (300D + sodium nitroprussiate—SNP); 500 mg L^−1^ N-primed with PEG water deficit induction + Nitric oxide scavenger (500D + cPTIO) (**c**) Relative fluorescent intensity of the histochemical nitric oxide (NO) accumulation of the leaves and roots (**d**) Relative fluorescent units (RFUs) of the released NO after 2-h incubation in 7 μM DAF-FM. Values shown are the Mean ± SE (n ≥ 30 and n = 4, respectively). The different letters above the bars indicate significant differences by one-way ANOVA and Duncan’s test (*p* ≤ 0.05), and the small letters represent the statistics of the leaves and the capital letters represent the statistics of the roots.

To further investigate the mechanisms underlining the elevated nitrogen priming in alleviation of oxidative damage, we therefore focused on the nitric oxide signaling which has been reported to trigger antioxidant defense mechanisms and set up an in-lab experiment to investigate the process with the application of nitric oxide donor and scavenger. Nitric oxide (NO) accumulation occurred under the PEG-induced water deficit conditions for elevated nitrogen primed plant roots and leaves (500D), and plants without elevated nitrogen priming but supplied with a nitric oxide donor, sodium nitroprusside (SNP) (300D + SNP) (Figure 5). In contrast, the released NO and histological NO accumulation remained low under PEG-induced water deficit in the plant without elevated N priming (300D) and plants-primed with 500 mg L^−1^ N but supplied with a NO scavenger, 2-(4-carboxyphenyl)-4,4,5,5-tetramethylimidazoline-1-oxyl-3-oxide (cPTIO) (500D + cPTIO) (Figure 5).

**Figure 6:**
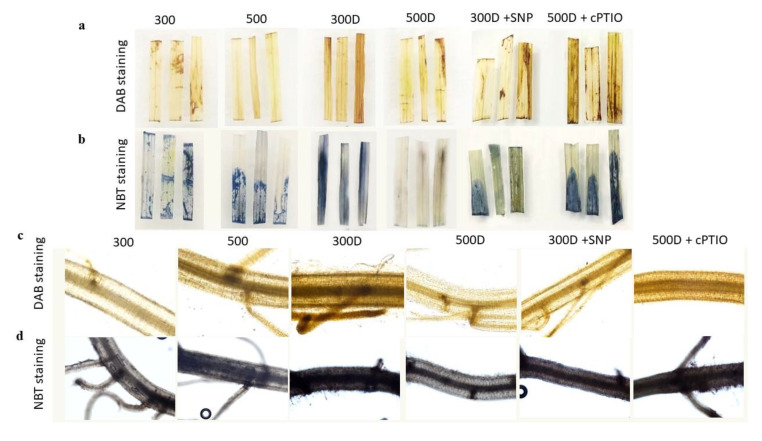
Reactive oxygen species accumulation responses. Hydrogen peroxide accumulation detected by 3,3’-diaminobenzidine (DAB) staining of (**a**) leaves and (**c**) roots. Superoxide radical accumulation detected by nitro blue tetrazolium (NBT) staining of (**b**) leaves and (**d**) roots of rice plants after 3-day growth in the different NO-associated treatments.

**Figure 7:**
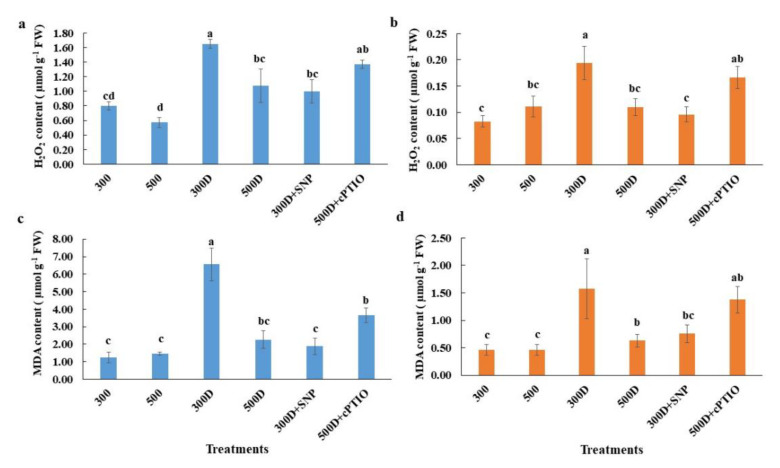
Quantitative ROS accumulation and lipid peroxidation responses: quantitative hydrogen peroxide accumulation in (**a**) leaves and (**b**) roots, and malondialdehyde (MDA) content of (**c**) leaves and (**d**) roots of rice plants after 3-day growth in the different NO-associated treatments. Values shown are the Mean ± SE (n = 4). The different letters above the bars indicate significant differences by one-way ANOVA and Duncan’s test (*p* ≤ 0.05).

Additionally, H_2_O_2_ content and staining showed low ROS levels in roots and leaves in plants without PEG treatment (control condition) (Figure 6 and Figure 7a,b). The accumulation of ROS and lipid peroxidation were induced by PEG as shown in 300D. However, ROS and MDA content remained low in 500D plants and 300D +SNP (Figure 6 and Figure 7), while the endogenous NO and released NO were more pronounced in these treatments (Figure 6 and Figure 7). The cPTIO also inverted elevated nitrogen priming effects, leading to ROS accumulation and lipid peroxidation (Figure 6 and Figure 7).

## 3. Discussion

Nitrogen is an essential nutrient that plays the main role in leaf development. In many cases, high nitrogen application induced leaf area increment and total plant transpiration increment while N also increases root growth, leading to the added water and nutrient uptakes to support the increased transpiration and maintain high photosynthetic activity and chloroplast functions during water deficit conditions [23]. However, high nitrogen priming during a short period of nitrogen application effects were first investigated here (prior to drought episodes). We found rice plants primed with 500 mg L^−1^ N prior to water deficit conditions maintained higher photosynthetic rates (Figure 1a) but did not alter the transpiration rate (Figure 2c), resulting in a dramatic increase in instantaneous water use efficiency (Figure 2d). Elevated nitrogen priming in our studies also induced plant responses similar to the high soil-incorporated nitrogen application [20,21]. Haefele et al. [24] and Zhong et al. [25] both suggested the use of high nitrogen fertilizer increases water-use efficiency, drought tolerance and survival rates for C3 and C4 crops. The improvement of photosynthetic capacity mainly depended on the activity of ribulose-1,5-bisphosphate carboxylase (Rubisco). Around 15 to 30% of leaf nitrogen are invested in Rubisco [26]. The high nitrogen levels induced higher Rubisco content, which was generally degraded during stress episodes [27]. The elevated N-primed rice might delay the degradation of Rubisco and maintain a high photosynthetic rate under water deficit conditions, as well as less sensitive stomatal behavior [24,25]. The maintenance of relative water content in elevated N-primed plants may be explained by the accumulation of NO as shown in Li et al. 2013 where the NO donor could have induced fructans accumulation, which resulted in plant cell water content regulation [12].

On the other hand, Gao et al. 2019 indicated that low nitrogen priming levels reduced the transpiration rate, stomatal conductance and maintained a higher leaf relative water content to mitigate drought-induced damage [28]. We also found slightly lower transpiration rates (Figure 2c) and significantly higher leaf relative water content (Figure 2a) in 100 mg L^−1^-N primed plants. Low nitrogen priming may also have induced lower chlorophyll and protein content, slowing down the process of stress recovery [29].

Although elevated N (500 mg L^−1^-N) priming benefits the tolerance of rice plants, excessive N (1000 mg L^−1^-N) priming leads to stress symptoms, like decreasing photosynthetic activity (Figure 1a), lower RWC (Figure 2a) and higher electrolyte leakage (Figure 3c), compared to plants primed with 500 mg L^−1^ N under control conditions. Excessive nitrogen application can also generate an imbalance in the carbon/nitrogen ratio which causes mature leaf senescence, leading to yield reduction after drought episodes [30]. Excessive nitrogen has impacts on the antioxidant defense systems in wheat [31], and in our study (Figure 4). Many nonenzymatic antioxidant molecules, such as GABA, 4-hydroxybenzoyl-choline and several phenolic compounds were downregulated in the leaves of rice plants grown under excessive N, which led to the overaccumulation of ROS [31].

Redox imbalance under water deficit conditions is accountable for significant plant cell damage. The antioxidant induction system is a viable approach to alleviate cellular damage and a higher tolerability of plants to water deficit conditions [10,32,33]. We observed enhanced antioxidant enzyme activity in 500 mg L^−1^ N primed rice (Figure 1a and Figure 2a). Perhaps the one-day elevated N priming was sufficient to activate antioxidant systems. Five-hundred mg L^−1^ N priming reduced membrane damage as demonstrated by the lower electrolyte leakage (Figure 3c) and MDA content (Figure 7d), which were supported by increasing SOD and APX activities (Figure 4a,c). Antioxidant defense systems act as drought tolerance mechanisms in many crop species, including rice, creeping bentgrass (*Agrostis stolonifera* L.), wheat (*Triticum aestivum* L.) seedlings and peanuts (*Arachis hypogaea* L.) [34,35,36,37,38].

The antioxidant defense system that controls ROS status in plant cells has also been reported to be regulated by the level of reactive nitrogen species (RNS) from both endogenous production and exogenous application [8,9,35,36,39]. Cai et al. 2015 [9] found that the overexpression of rat neuronal NO synthase (nNOS) in rice enhanced drought and salt tolerance of rice with a higher NOS activity and the accumulation of NO, together with a reduction in H_2_O_2_ accumulation, electrolyte leakage and MDA content. Using NO donors, SNP as a seed priming solution and foliar spray has previously induced rice drought tolerance in rice [13]. Because NO acts as a signaling molecule it can enhance antioxidant capacity thus reducing oxidative damage [13]. The 500 mg L^−1^ N leaves and roots accumulated higher endogenous NO and released NO, which was inhibited by the NO scavenger, cPTIO (Figure 5b–d). The level of NO accumulation was close to that of the normal N-primed (300 mg L^−1^ N) plants supplied with 1 mM SNP (Figure 5b,d). In contrast, 500 mg L^−1^ N primed plants possessed less O_2_^•−^ and H_2_O_2_ accumulation evidenced by the NBT, DAB staining and H_2_O_2_ quantification (Figure 6 and Figure 7a,b). We measured only O_2_^•−^ and H_2_O_2_ because these two species have been reported to directly interact with NO [40,41]. Adding cPTIO may mitigate such effects (Figure 6 and Figure 7a,b). Control plants (300 mg L^−1^ N) had less O_2_^•−^ and H_2_O_2_ further suggesting a relationship between elevated N priming and increased NO (Figure 6 and Figure 7a,b). This phenomenon might be explained by the fact that NO in plants can be generated by NO synthase (NOS), nitrate reductase (NR) and xanthine oxidoreductase [10]. When priming rice with elevated N, it induced NR activity, promoting greater nitrite levels (precursors for both NO production and N assimilation [42]. Higher NO levels in elevated N-primed rice promoted the antioxidant defense system, causing less accumulated ROS and less oxidative damage while maintaining photosynthetic functions. NO acted as a potent inhibitor of lipid peroxidation by scavenging lipid alcoxyl (LO˙) and peroxyl (LOO˙) radicals [43]. NO also directly quenches the ROS, such as superoxide radical (O_2_^•−^) [44], limits oxidative damage and prevents the onset of cell death [45]. Moreover, NO maintained membrane fluidness, cell wall relaxation, cell enlargement and plant growth under stressful conditions [11,42]. Therefore, elevated nitrogen priming demonstrated a water deficit tolerance through NO-mediated antioxidant defense mechanisms.

## 4. Materials and Methods

### 4.1. Plant Material and Greenhouse Growth Conditions

Seeds of Thai rice (*Oryza sativa* L. subspecies indica cv. Pathumthani 1) were germinated on moist germination paper for 10 d at 28 °C in the dark. Seedlings were transplanted into 2-L pots filled with 1:1 vermiculite: perlite. The seedlings were grown in the greenhouse with day lengths of 11–12 h and daily average temperatures of 35 ± 4/24 ± 7 °C day/night and 700–1500 µmol m^−2^ s^−1^ mid-day photosynthetically active radiation (PAR). Plants were fertilized every other day with (N 300 mg L^−1^ equal in nitrate and ammonium forms), P 20 mg L^−1^, K 75 mg L^−1^, Ca 25 mg L^−1^, Mg 17 mg L^−1^, S 55 mg L^−1^, Fe 3.30 mg L^−1^, Mn 0.50 mg L^−1^, Zn 0.05 mg L^−1^, Mo 0.01 mg L^−1^, Cu 0.02 mg L^−1^) for 10 weeks until the plants were one week before panicle initiation. Then, different concentrations of nitrogen (N) fertilizer were applied by using N 100 mg L^−1^ (equal in nitrate and ammonium forms), P 20 mg L^−1^, K 75 mg L^−1^, Ca 25 mg L^−1^, Mg 17 mg L^−1^, S 55 mg L^−1^, Fe 3.30 mg L^−1^, Mn 0.50 mg L^−1^, Zn 0.05 mg L^−1^, Mo 0.01mg L^−1^, Cu 0.02 mg L^−1^ as base fertilizer and added ammonium nitrate (NH_4_NO_3_) to reach target concentrations of 300, 500 and 1000 mg L^−1^ N and used double volume of the field capacity of the pots to wash the previous nitrogen fertilizer. Water deficit stress was applied by withholding water for ~7 d when visual stress symptoms (i.e., leaf rolling) appeared (10–15% relative soil water content) [7], when the gas exchange parameters were measured and the leaf samples were taken for biochemical assays. The leaves were collected between 9.00 am and 11.00 am and immediately frozen in liquid nitrogen and kept at −80 °C until use.

### 4.2. Gas-Exchange Measurements

Net photosynthetic rate and stomatal conductance were measured with the photosynthesis system ADC LCi-SD (BioScientific, UK). The measurements were conducted under 900 ± 50 μmol m^−2^ s^−1^ light intensity and 380 ± 10 μmol mol^−1^ CO_2_ surrounding the leaf (Ca) at 32 ± 2 °C. To calculate instantaneous transpiration, the transpiration rate was divided by the net photosynthetic rate.

### 4.3. Leaf Area

After one-day post recovery, all remaining leaves were removed and all surface dust was wiped away prior to measuring the area with an LI-3100C area meter (LI-COR, Lincoln, NE, USA) in square centimeters.

### 4.4. Relative Water Content (RWC) of Leaves

RWC was determined according to Gao et al. 2019 [28]. Leaves were weighed immediately to obtain fresh weight (FW), soaked in water overnight in the dark and weighed again to obtain turgid fresh weight (TW), and then dried at 75 °C until constant weight (DW). RWC was then calculated as follows:

RWC = (FW−DW)/(TW−DW)(1)

### 4.5. Electrolyte Leakage (EL)

The electrolyte leakage measurement was adjusted based on the method described previously by Cai et al. 2015 [9]. After placing six 1 cm^2^ leaves from each treatment into a 50-mL-tube with 20 mL distilled deionized water, the tubes were shaken and electro-conductivity (*EC_i_*) was immediately measured with a pH/cond meter (WTW, inoLab, Germany). After soaking the leaves for 12 h, the second conductivity was measured (*EC_f_*). Then, the leaves were boiled for 1 h and the total electro-conductivity was measured (*EC_t_*). The electrolyte leakage was calculated as follows:

(2) $$\%\;\mathrm{EL}\;=\;\left(\frac{EC_{f}-EC_{i}}{EC_{t}-EC_{i}}\right)\times 100$$

### 4.6. Determining SOD, CAT and APX Activities

Enzymes were extracted by the modified method of Umnajkitikorn et al. 2013 [46]. The frozen leaves were ground in liquid nitrogen with a mortar and pestle. Two hundred fifty mg of the leaf powder were homogenized in 1 mL of the extraction buffer containing 50 mM potassium phosphate buffer (pH 7.8), 0.1 mM disodium EDTA, 1 mM ascorbic acid and, 2% PVPP (*w*/*v*). The homogenate was centrifuged at 15,000× *g* for 20 min at 4 °C. The supernatant was collected for SOD, CAT and APX activity assays.

SOD activity was assayed based on the method described by Beauchamp and Fridovich 1971 [47] and Vaidyanathan et al. 2003 [48]. One point two milliliters of the reaction mixture, containing 50 mM sodium phosphate buffer (pH 7.8), 10 mM EDTA, 1 mM NBT, 5 mM L-methionine, 0.2 mM riboflavin and mixed with 80 µL of extracts. Each reaction was carried out at 25 ± 2 °C under a light intensity, which is sufficient to increase absorbance of 0.110/10 min (in the absence of the enzyme) for 30 min. Two hundred microliters of the reactions were taken to Nunc microwell 96-well plate (Thermo ScientificTM, Shanghai, China) for the absorbance at 560 nm (A560), using spectrophotometer (Biotex, Epoch, Winooski, VT, USA). The nonirradiated reaction mixture served as a blank and was deducted from A560. One unit of SOD activity was defined as the amount of enzyme required to inhibit the reduction of NBT by 50%.

CAT activity was assayed based on the method of Sunohara and Matsumoto 2004 [49]. One hundred and ninety microliters of the assay mixture contained 20 mM H_2_O_2_ in 50 mM potassium phosphate buffer (pH 7.0), 0.1 mM disodium-EDTA and 10 µL of extracts. The reactions were carried in Nunc 96 well UV transparent plate (Thermo ScientificTM, Vantaa, Finland). The absorbance was measured at 240 nm with a UV/VIS spectrophotometer (Biotex, Epoch, Winooski, VT, USA). The enzyme activity was defined as the amount of H_2_O_2_ decomposed min^−1^ mg protein^−1^. The molar coefficient of H_2_O_2_ (E) is 39.4 M^−1^ cm^−1^) at 240 nm.

APX activity was assayed based on the method of Sunohara and Matsumoto 2004 [49]. One hundred and eighty microliters of the assay mixture contained120 µL of 50 mM potassium phosphate buffer (pH 7.0), 20 µL of 1 mM EDTA, 20 µL of 5 mM L-ascorbic acid, 20 µL of 1 mM H_2_O_2_ and 20 µL of extracts. The subsequent decrease in ascorbic acid was observed at 290 nm (E = 2.8 mM^−1^ cm^−1^) with a UV/VIS spectrophotometer (Biotex, Epoch, Winooski, VT, USA). The enzyme activity was defined as the amount of ascorbic acid (ASA) which decomposed min^−1^ mg protein^−1^.

### 4.7. Protein Quantification

The Bradford assay [50] was used for protein quantification using bovine serum albumin as the standard.

### 4.8. The RNS and ROS Experiment

Rice seeds (*Oryza sativa* subspecies indica cv. Pathumthani 1) were germinated and grown for 5 d at 25 °C in darkness. Seedlings were transplanted into 9-cm pots filled with vermiculite 4 plants/pot and grown in the growth room with 12 h/12 h light/dark cycles and 25/20 °C day/night cycles for 3 weeks with fertilizer containing 300 mg L^−1^ N. Then, the plants were randomly divided into 6 groups and transferred to glass tubes with the following treatments: (1) 300 mg L^−1^ N (300 C); (2) 500 mg L^−1^ a day and switched back to the 300 mg L^−1^ (500 C); (3) 300 mg L^−1^ N + gradually increased polyethylene glycol (PEG) 6000 (300 D); (4) 500 mg L^−1^ a day and switched back to the 300 mg L^−1^ + gradually increased PEG (500 D); (5) 300 mg L^−1^ N + PEG 6000 + nitric oxide donor, 1 mM SNP (300 D + SNP); (6) 500 mg L^−1^ N + PEG 6000 + 3 mM of Nitric oxide scavenger, 2-(4-carboxyphenyl)-4,4,5,5-tetramethylimidazoline-1-oxyl-3-oxide (cPTIO) (500 D + cPTIO). After 3 days under each treatment, the seedlings were harvested for further ROS and RNS analyses.

### 4.9. NO Determination

To histologically detect NO, the leaf and root segments were soaked for 1 h with 20 μM 4-amino-5-methylamino-2′,7′-difluorescein diacetate (DAF-FM DA; Sekisui Medical, Tokyo, Japan). The epifluorescence images were captured with an Eclipse 90i microscope (Nikon, Tokyo, Japan). The intensity of the fluorescent signal from at least 30 pictures of each treatment was quantified by using Image J software (by Wayne Rasband, NIH, MD, USA).

The concentration of NO released from the roots was assessed in leaf and root segments of the seedlings in each treatment after incubation in 7 μM DAF-FM for 2 h. The fluorescence intensity of the DAF-FM solution was measured as described in Fukudome et al. 2016 [51] but adapted by adding 100 µL of the solution into the 96 well black plate (SPL life sciences, Gyeonggi-do, Korea), then measured with a fluorescence spectrophotometer (Varioskan Lux, Finland) at 495 nm and 519 nm as excitation and emission wavelengths, respectively. The released NO was expressed as relative fluorescence units (RFUs) per fresh weight of leaves or roots.

### 4.10. Histochemical Detection of H_2_O_2_ and O_2_^•−^

H_2_O_2_ was detected in situ according to Fukudome et al. 2019 [39] and Signorelli et al. 2013 [52]. Detached leaves and roots were vacuum-infiltrated in the dark with 10 mM potassium phosphate buffer, and 0.1% (*w*/*v*) 3,3’-diaminobenzidine (DAB), at pH 7.8. Samples were incubated overnight in the dark. The leaf segments were boiled in 95% ethanol at 90oC to remove chlorophyll. The clear leaf segments were then photographed. The staining areas were calculated by Image J software (by Wayne Rasband, NIH, USA), in the red channel according to procedure described in [53]. Data were shown in Supplementary Figure S2. The detailed method of staining area identification is given in Supplementary file 2.

Superoxide radical (O_2_^•−^) was detected in situ essentially as described by to Fukudome et al. 2019 [39] and Signorelli et al. 2013 [52]. Detached leaves and roots were vacuum-infiltrated with 10mM potassium phosphate buffer, 0.1% (*w*/*v*) nitro blue tetrazolium (NBT), and 0.05% (*v*/*v*) Tween 20, pH 7.8. Treated samples were incubated overnight in the dark, cleared and photographed as described above. The staining areas were calculated by Image J software (by Wayne Rasband, NIH, MD, USA), in the blue channel according to the procedure described in [53]. Data are shown in Supplementary Figure S2.

### 4.11. H_2_O_2_ Quantification

The frozen leaves were ground and 5–10 mg of samples were extracted with 1 mL of 20 mM K_2_HPO_4_ (pH 6.5), homogenized at 1500 rpm for 5 min at 4 °C and then centrifuged for 5 min at 16,200× *g* at 4 °C. The H_2_O_2_ was colorimetrically quantified by Amplex Red detection assay (Thermo Fisher Scientific). The measurement was performed on the supernatant as described in Brumbarova et al. 2016 [54] with some modification of the incubation time from 30 min to 15 min. The assay solutions were analyzed at 560 nm with a UV/VIS spectrophotometer (Biotex, Epoch, Winooski, VT, USA). The standard curve was also generated with H_2_O_2_ concentration from 0–5 µM.

### 4.12. Malondialdehyde (MDA) Measurements

The youngest fully expanded leaves were homogenized with 5 mL of 50 mM potassium phosphate buffer pH 7.5 and centrifuged at 20,000× *g* for 25 min. For measurements of MDA concentration, 4 mL of 20% trichloroacetic acid containing 0.5% thiobarbituric acid were added to a 1 mL aliquot of the supernatant. The mixture was heated at 95 °C for 30 min, quickly cooled in ice and then centrifuged at 10,000× *g* for 10 min. The absorbance of the supernatant was measured at 532 nm. The results are shown as MDA content mg^–1^ FW, using malondialdehyde tetrabutylammonium salt as a standard (Sigma-Aldrich, Cat 63287, Singapore).

### 4.13. Statistical Analysis

The SPSS 25 statistical package was used for statistical analyses. The experiments were based on a randomized complete block design.

## 5. Conclusions

Elevated N priming at approximately 60% more than normal N level enhanced water deficit tolerance with NO-triggered antioxidant defense systems. The elevated N priming also supported the sustainability of photosynthetic activity and relative water contentof the leaves, together with the reduction of membrane damage. This approach has potential for in-situ investigation in aerobic rice fields with fertigation systems as a potential mitigating factor for enhancing drought tolerance in rice.

## Data Availability

Data available in a publicly accessible repository.

## Supplementary Materials

The following are available online at https://www.mdpi.com/2223-7747/10/2/381/s1, Supplementary file 1: Fertilizer Information and Formulation sheet. Supplementary file 2: Figure S1: Examples of the picture calibrated for staining area evaluation. Figure S2: The ROS staining area of tissue segments of rice plants after 3–day growth in the different NO-associated treatments; Procedure for staining area quantification by image J.
