# Supplementary material for: Elevated Nitrogen Priming Induced Oxinitro-Responses and Water Deficit Tolerance in Rice
Source: Plants (Basel). 2021 Feb 17;10(2):381. doi: 10.3390/plants10020381 (PMC7922565; doi:10.3390/plants10020381)
Supplement: Supplementary file 1 [file plants-10-00381-s001.zip › sup-20210211/Supplementary file 2.final.pdf]

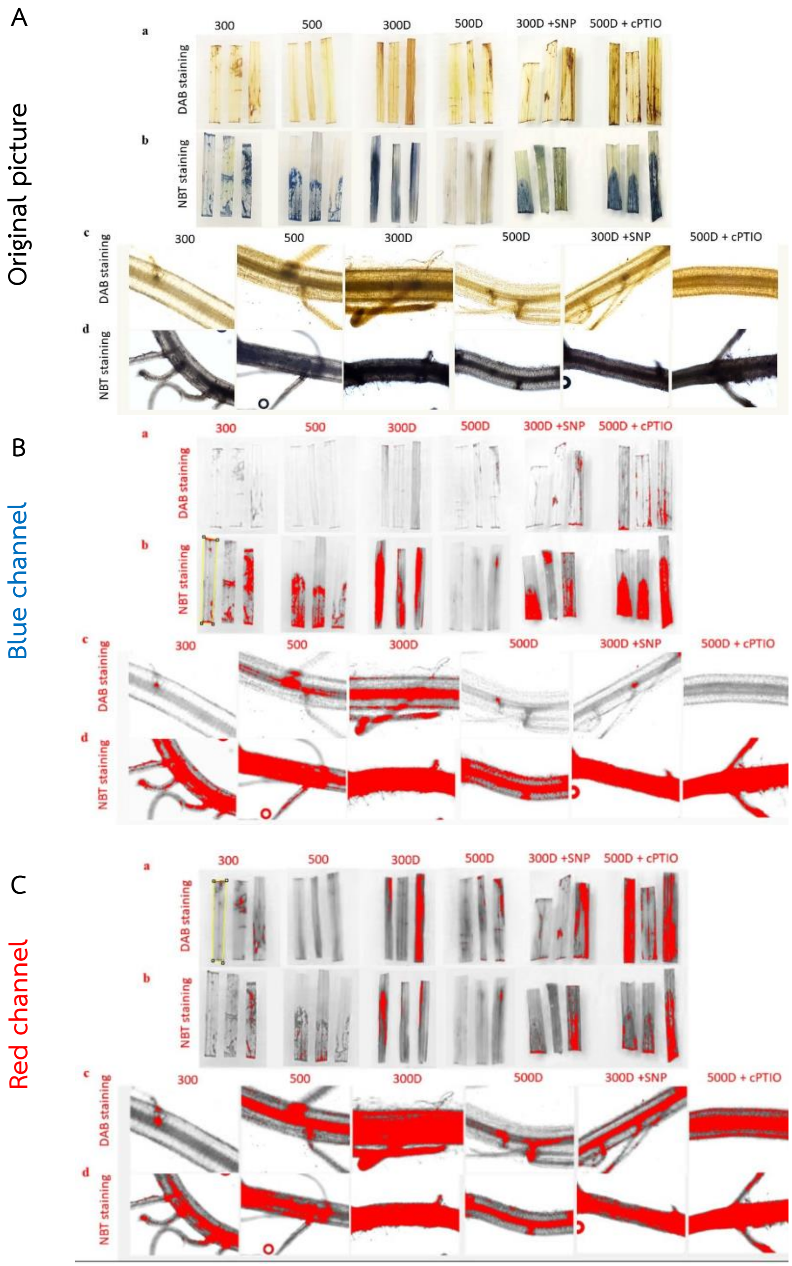

Figure S1. Examples of the picture calibrated for staining area evaluation. The red area in each sample segment (indicated by yellow lines) represents the staining area and was used to calculated the staining area percentage in Figure S2.

A) The RGB color of the original picture
B) The Blue channel of the image was used for the calculation of the percentage of the NBT staining area
C) The Red channel of the image was used for the calculation the percentage of the DAB staining area

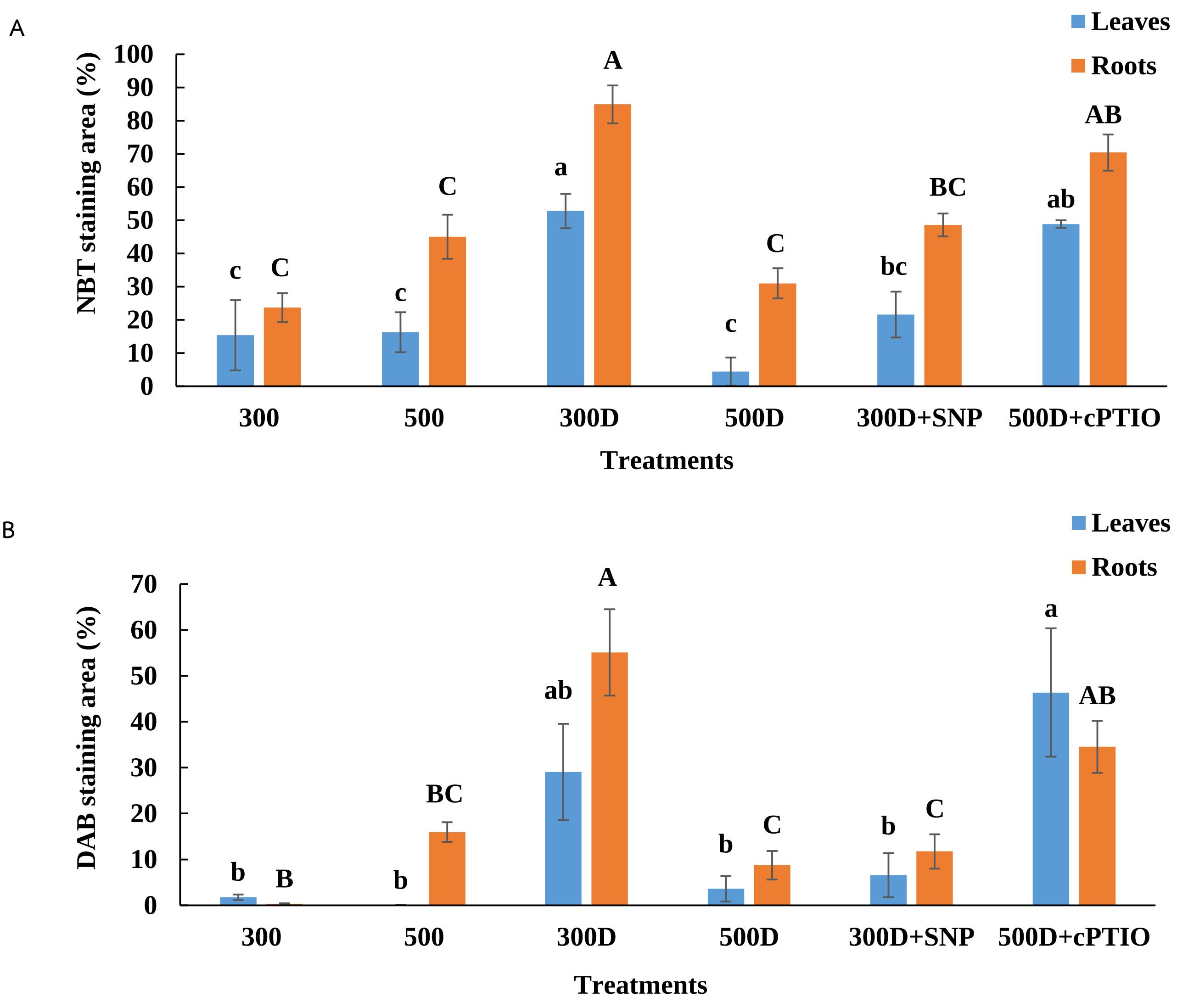

Figure S2. The ROS staining area of tissue segments of rice plants after 3-day growth in the different NO-associated treatments

A) The staining area of NBT stained leaf and root segments
B) The staining area of DAB stained leaf and root segments

Values are the Mean ± SE (n = 10). The different letters above the bars indicate significant differences by one-way ANOVA and Duncan's test ($P \leq 0.05$), the small letters represent the statistics of the leaves and the capital letters represent the statistics of the roots.

Procedure for staining area quantification by image J

1. Split the image into red, green and blue channels by going to the Image>Type>RGB Stack command
2. Adjust the image threshold of the desirable channel (Blue in the case of NBT staining and Red in the case of DAB staining) to match with the original RGB picture by going to Image>Adjust>Threshold
3. Use the Image>Stacks>Make Montage command to view all three channels at the same time.
4. Set the required information from the measurement by going to Analyze>Set Measurements dialog and checking "Area", "Area Fraction", "Limit to Threshold" and "Display Label".
5. Select the area of whole leaf/root segment by the selection tools 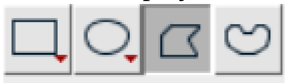 (in this case we used polygon selections).
6. Press Ctrl + M (Analyze>Measure) to measure the staining area and the % Area (Area Fraction)
7. In the "Results" window, select all data and copy to Microsoft Excel for further analyses.
